# Variation in plasma calcium analysis in primary care in Sweden - a multilevel analysis

**DOI:** 10.1186/1471-2296-11-43

**Published:** 2010-05-30

**Authors:** Sofia Dalemo, Per Hjerpe, Henrik Ohlsson, Robert Eggertsen, Juan Merlo, Kristina Bengtsson Boström

**Affiliations:** 1 Dept of Public Health and Community Medicine/Primary Health Care, Sahlgrenska academy Gothenburg University, PO Box 454, SE-405 30 Gothenburg, Sweden; 2 R&D Centre Skaraborg Primary Care, Storgatan 18, SE-541 30 Skövde, Sweden; 3 Unit of Social Epidemiology, CRC, Faculty of Medicine, Lund University, Skåne University Hospital, entrance 72, SE-205 05 Malmö, Sweden

## Abstract

**Background:**

Primary hyperparathyroidism (pHPT) is a common disease that often remains undetected and causes severe disturbance especially in postmenopausal women. Therefore, national recommendations promoting early pHPT detection by plasma calcium (P-Ca) have been issued in Sweden. In this study we aimed to investigate variation of P-Ca analysis between physicians and health care centres (HCCs) in primary care in county of Skaraborg, Sweden.

**Methods:**

In this cross sectional study of patients' records during 2005 we analysed records from 154 629 patients attending 457 physicians at 24 HCCs. We used multilevel logistic regression analysis (MLRA) and adjusted for patient, physician and HCC characteristics. Differences were expressed as median odds ratio (MOR).

**Results:**

There was a substantial variation in number of P-Ca analyses between both HCCs (MOR_HCC _1.65 [1.44-2.07]) and physicians (MOR_physician _1.95 [1.85-2.08]). The odds for a P-Ca analysis were lower for male patients (OR 0.80 [0.77-0.83]) and increased with the number of diagnoses (OR 25.8 [23.5-28.5]). Sex of the physician had no influence on P-Ca test ordering (OR 0.93 [0.78-1.09]). Physicians under education ordered most P-Ca analyses (OR 1.69 [1.35-2.24]) and locum least (OR 0.73 [0.57-0.94]). More of the variance was attributed to the physician level than the HCC level. Different mix of patients did not explain this variance between physicians. Theoretically, if a patient were able to change both GP and HCC, the odds of a P-Ca analysis would in median increase by 2.45. Including characteristics of the patients, physicians and HCCs in the MLRA model did not explain the variance.

**Conclusions:**

The physician level was more important than the HCC level for the variation in P-Ca analysis, but further exploration of unidentified contextual factors is crucial for future monitoring of practice variation.

## Background

Primary hyperparathyroidism (pHPT) is a common disease that often remains undetected and causes severe disturbance especially in postmenopausal women. Therefore, national recommendations promoting early pHPT detection by plasma calcium (P-Ca) have been issued in Sweden [1,2]. In this study we aimed to investigate variation of P-Ca analysis between physicians and health care centres (HCCs).

pHPT is a potentially serious condition leading to increased morbidity and mortality from cardiovascular disease [3] and cancer [4]. Although mild disease might not increase the risk [5]. PHPT gives raised plasma calcium (P-Ca) and because of the vague symptoms, pHPT is difficult to detect without an analysis of P-Ca.

Even though previous studies indicate that the frequency of P-Ca analyses differs between health care centres (HCC) [6] the understanding of the relative importance of the different levels (patients, physicians, HCCs) for these differences is limited. However, in a study from New Zealand where a defined clinical situation was presented to GPs, it was shown that inherent characteristics of the physicians more than the patients clinical situation determined which laboratory tests were ordered [7]. A study from the Netherlands, without patient characteristics, found a regional variation in laboratory testing and that factors at both the physician and HCC level influenced the inclination to order tests [8].

The aim of this study was to investigate the relative importance of the different levels in the health care organization for P-Ca analyses using the Skaraborg Primary Care Database (SPCD). Identification of factors contributing to the variation can be of relevance for planning interventions for an optimal frequency of P-Ca analyses and for evaluating the national recommendations.

## Methods

### Study population

Skaraborg is a rural area in Sweden and comprised 255 758 inhabitants in 2005. The public primary care is a part of the Västra Götaland region and serves 97% of the population (n = 247 985). All the HCCs (n = 24) use the same computerised medical record, ProfDoc Journal III (PDIII ProfDoc AB: Uppsala) facilitating data extraction. SPCD has been created containing encrypted data from patients and caregivers from all HCCs. The database contains patients' age, sex, diagnoses, laboratory analyses, and drug prescriptions. The HCCs' laboratory facilities are accredited by SWEDAC (the Swedish Board for Accreditation and Conformity Assessment). The validity of the information in the database has recently been audited and judged to be mostly appropriate but varying with type of diagnosis [9]. All 154 629 individuals that attended any of the 24 HCCs during 2005 are included in the analysis. The local ethics committee at Gothenburg University approved the study (255-09).

### Study procedure and assessment of variables

The outcome variable was P-Ca analyses during 2005 (yes/no). Sex of the patient and P-Ca analyses during 2004, were included as explanatory variables. We also selected ICD-10 coded diagnoses and symptoms associated with pHPT [10] . A risk score for a P-Ca analysis was created with stepwise logistic regression [11] based on age, concomitant diagnosis and drug treatment, in order to control for confounding factors. The risk score was divided in quintiles, patients with the lowest risk of P-Ca analyses (group 1) were used as reference. The main characteristics included in the risk score are listed in Table 1 A more detailed description can be found in additional file 1.

**Table 1:** Examples of diagnoses with strong influence of the chance of having a plasma calcium analyses the risk score equation. Total number of patients 154 629.

*Title*	*All the positive diagnoses in the stepwise regression*	*ICD-10 Codes*	*Odds ratio*	*95% *	*CI*	*Number of P-Ca analysis*
Neoplasms	Sarcoidosis	D86	8.4	3.3	21.4	21
						
Endocrine disorders	Nontoxic goitre	E04	3.1	2.2	4.5	177
	Other disorders of thyroid	E07	3.6	2.0	6.4	63
						
Mental disorders	Unspecified dementia	F03	2.5	2.0	3.0	523
	Depressive episode	F32	2.3	2.0	2.5	3196
	Anxiety disorder	F41.9	1.7	1.5	2.0	1438
	Nonorganic sleeping disorders	F51	1.5	1.3	1.7	1636
						
Diseases of the circulatory system	Essential hypertension	I10	1.8	1.7	1.9	12867
	Atrial fibrillation and flutter	I48	1.5	1.3	1.6	1792
	Heart failure	I50	1.7	1.5	1.9	1937
						
Diseases of the digestive system	Constipation	K59.0	1.7	1.4	2.0	730
						
Diseases of the musculoskeletal system	Other artritis and rheumatism unspecified	M13	2.4	1.9	2.9	577
	Myalgia	M79.1	1.8	1.6	2.0	3749

***Symptoms***	Abnormal blood-pressure reading, without diagnosis	R03.0	2.7	2.3	3.3	580
	Polyuria	R35	2.0	1.5	2.7	355
	Headache	R51	2.5	2.2	2.9	1392
	Malaise and fatigue	R53	6.5	5.9	7.2	2261

**Contact with health services**	General medical examination	Z00.0	3.3	3.0	3.7	1911
	Worried well	Z71.1	2.5	2.2	2.8	1732

***Drug***	Calcium and vitamin D supplements		3.3	2.1	5.1	2938
	Thiazide diuretics		1.3	1.2	1.4	8305

P-Ca = plasma calcium

95% CI = 95% credible interval

The physicians were categorised according to sex and title. GP and locum were also dichotomised at 46 year. GPs, 46 years or older, were used as reference in the analysis. As only six doctors among house officers and preregistration house officers were above 45 years, they were not dichotomised.

The HCCs had different standardised group analyses, for instance analyses of electrolytes, hypertension check ups and diagnosing dementia, in which P-Ca was included. We categorised HCCs as having none, 1-2, and ≥ 3 standardised groups including P-Ca. The HCCs having no group analyses were used as reference.

### Statistical analysis

**Figure 1:**
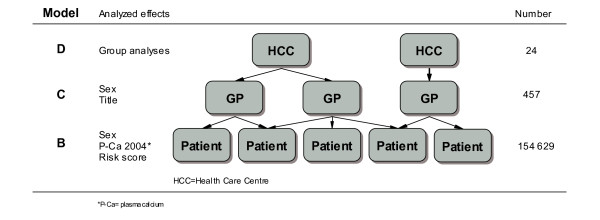
**Multilevel model employed in plasma calcium analyses in primary care County of Skaraborg, Sweden**. A three-level logistic regression model including health care centre (HCC), physician and patient levels. At each level the analysed effect and the number of elements are described. The arrows depict the nesting of patients within physicians and physicians within HCCs and crossing arrows the multiple memberships.

We used multilevel logistic regression analysis (MLRA) to estimate the odds of patients being ordered a P-Ca analysis, as the data had a hierarchical structure (i.e., patients nested within physicians nested within HCCs) [12,13]. As one patient could attend several physicians and several HCCs, we used a multiple membership model (Figure 1) [14] . The weights were constructed according to number of visits to a certain physician/HCC during our study period.

We developed four consecutive models. Model A included the random parameters (physicians and HCCs), in order to partition the variance at different levels. Model B included the patient characteristics, model C the patient and physician characteristics and model D the patient, physician and HCC characteristics. In this way we could investigate whether the contextual characteristics explained the residual variation at the physician and HCC levels.

In the fixed-effects part of the MLRA, we calculated odds ratios (OR) and their 95% credible intervals (95% CI). In the random-effects part of the MLRA, we obtained the variance at the physician and HCC levels. In order to quantify the importance of the different levels in the analysis we calculated the median odds ratio (MOR) [15,16]. The MOR translates the variance into the widely used OR scale, and can thereby be directly compared with the ORs of individual or area variables. In very simple terms, the MOR could be interpreted as how much a patient's odds of being ordered a P-Ca analysis would (in median) increase if this patient moved to a physician/HCC with higher odds of ordering a P-Ca analysis. A MOR of one indicates that there are no differences between physicians/HCCs in their odds of ordering P-Ca analysis. The larger the differences between physicians/HCCs are, the larger the MOR will be. The MOR_physician+HCC _is applicable to patients that visited only one physician during the study period. Parameters were estimated by MCMC methods [17] in the MLwiN 2.02 software [18].

## Results

Overall 5.8% of the inhabitants in Skaraborg and 9% of the patients (11% of the women and 8% of the men) attending the HCCs had a P-Ca analysis. The mean age of the patients with P-Ca analysis was 62 years compared to 45 years for patients with no P-Ca analyses. At the different HCCs the number of standardised group analyses including P-Ca analyses varied from zero to seven. The locums were most numerous shown in Table 2, but had short periods of attendance.

**Table 2:** Staffing of physicians at health care centres, characteristics of physicians and number of patients visits and frequency of plasma calcium analyses per title in the county of Skaraborg during 2005.

	Physicians						Physicians/HCC				Patients visits	
	Total number	% women	Mean age (year)	SD	Median age (year)	Range age (year)	Mean	SD	Median	Range	Total number	With P-Ca test %
Preregistration house officer	51	39	31	5.6	29	26-48	2.2	2.7	1	0-7	21 424	11
House officer	68	69	35	6.0	34	26-50	3.0	1.9	3	0-9	35 712	11
GP < 46 year	39	41	38	3.6	35	32-45	1.7	1.4	2	0-4	45 491	10
GP ≥ 46 year	85	32	55	8.6	52	46-67	3.7	1.6	4	1-6	92 109	8
Locum < 46 year	112	17	36	5.4	36	27-45	6.2	8.9	3	0-36	23 153	7
Locum ≥ 46 year	102	17	55	11.5	46	46-76	6.5	9.2	2	0-32	23 573	8

**Total**	457	32	43	11.6	41	26-76	23.3	18.1	18	2-85	241 529	9

GP = general practioner

HCC = health care centre

P-Ca = plasma calcium

### The multilevel model

**Figure 2:**
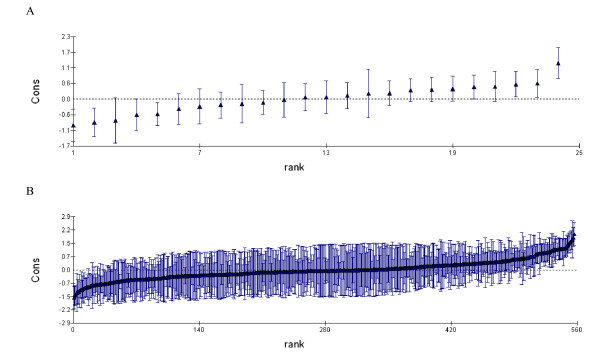
**Plasma calcium analyses per physician and HCC in primary care in county of Skaraborg, Sweden**. A. percent plasma calcium analyses per physician. B. percent plasma calcium analyses per health care centre (HCC)

There was a substantial variation in number of P-Ca analyses between HCCs and physicians. The four models used in the analyses are shown in Table 3. In model A the MOR_physician+HCC _indicated that for a patient changing both GP and HCC, to a GP and HCC with higher odds for a P-Ca analysis, the odds would in median increase by 2.31. The physician level, MOR_physician _= 1. 95 (95% CI: 1. 85-2.08) contributed more than the HCC level, MOR_HCC _= 1.65 (95% CI: 1.44-2.07). Figure 2 shows the residuals for physicians (Panel A) and for HCCs (Panel B) from the multilevel analysis.

**Table 3:** Multi-level logistic regression analysis of plasma calcium analyses in primary care in the county of Skaraborg, Sweden

	Model A	Model B	Model C	Model D
**Fixed effects**	OR (95%CI)	OR (95%CI)	OR (95%CI)	OR (95%CI)
*Patient*				
Female	-	REF	REF	*REF*
Male	-	*0,80 (0,77-0,83)*	*0,80 (0,77-0,83)*	*0,80 (0,77-0,83)*
				
P-Ca test 2004	-	*1,44 (1,37-1,51)*	*1,44 (1,36-1,51)*	*1,44 (1,37-1,51)*
				
Risc score				
Group 1	-	REF	REF	REF
Group 2	-	*2,40 (2,15-2,70)*	*2,43 (2,18-2,71)*	*2,40 (2,14-2,68)*
Group 3	-	*4,51 (4,08-5,04)*	*4,56 (4,13-5,06)*	*4,51 (4,08-4,97)*
Group 4	-	*8,92 (8,11-9,87)*	*9,01 (8,14-9,96)*	*8,91 (8,12-9,78)*
Group 5	-	*25,8 (23,5-28,5)*	*26,1 (23,7-28,8)*	*25,8 (23,5-28,4)*
*Doctor*				
Female	-	-	REF	REF
Male	-	-	0,93 (0,78-1,09)	0,95 (0,78-1,24)
Preregistration house officer	-	-	1,48 (1,00-2,00)	*1,51 (1,07-2,05)*
House officer	-	-	*1,69 (1,35-2,24)*	*1,57 (1,26-2,09)*
GP < 46 year	-	-	*1,30 (1,02-1,76)*	1,16 (0,93-1,60)
GP ≥ 46 year	-	-	REF	REF
Locum < 46 year	-	-	0,84 (0,61-1,08)	0,78 (0,58-1,03)
Locum ≥ 46 year			0,73(0,57-0,94)	0,69(0,51-0,89)
*HCC*				
Number groups include P-Ca				
Group 1	-	-	-	REF
Group 2	-	-	-	*2,59 (1,56-3,53)*
Group 3	-	-	-	*2,79 (1,25-5,09)*
				
**Random effects**	Variance (95%CI)	Variance (95%CI)	Variance (95%CI)	Variance (95%CI)
HCC (Intercept)	0,28 (0,15-0,58)	0,32 (0,16-0,67)	0,32 (0,18-0,66)	0,36 (0,16-0,80)
MOR_*HCC*_	1,65 (1,44-2,07)	1,71 (1,47-2,18)	1,72 (1,49-2,17)	1,77 (1,48-2,34)
Physician (Intercept)	0,49 (0,41-0,59)	0,59 (0,50-0,71)	0,52 (0,43-0,62)	0,52 (0,43-0,63)
MOR_*Physician*_	1,95 (1,85-2,08)	2,09 (1,96-2,24)	1,98 (1,87-2,12)	1,99 (1,88-2,13)
HCC and Physician (Intercept)	0,77	0,91	0,84	0,88
MOR_*HCC*+*Physician*_	2,31	2,48	2,4	2,45
DIC	89 550	76 438	76 427	76 427

Figure in italics are significant at 0.05 level

P-Ca = plasma calcium

95% CI = 95% credible interval

MOR = median odds ratio. OR = odds ratio

### Model B, C and D

Model B illustrates that female sex and increased number of diagnoses in the risk score was associated with higher propensity of a P-Ca analysis However, the inclusion of other patient characteristics did not explain the variance at the physician or HCC level. Model C illustrates that house officers, preregistration house officer and younger GPs ordered more P-Ca analysis compared to older GPs. Locums, regardless of age, ordered fewer P-Ca analyses. There were no differences between male and female physicians. Inclusion of the physician characteristics did not explain the variance in model A. Model D illustrates that a high number of standardised group analyses were associated with a high number of P-Ca analyses. The inclusion of HCC characteristics did not explain the variance at the higher levels.

Including all explanatory variables and controlling for confounders, a patient changing both GP and HCC, from low to high odds for P-Ca analysis, the odds for a P-Ca analysis would in median increase by 2.5 times, MOR_physician+HCC _2.45.

## Discussion

The main finding of this study was that the ordering of P-Ca analyses was influenced by factors both at the physician and at the HCC level, with the physician level being more important than the HCC level. Theoretically, if a patient were able to change both GP and HCC, the odds of undergoing a P-Ca analysis would in median increase by 2.45 times. Including compositional and contextual characteristics in the model did not explain the variance at the higher levels.

Overall 5,8% of the inhabitants underwent a P-Ca analysis, which is comparable with an earlier study from Skaraborg (6,1%) [6] and two-fold compared with a study from primary care in southern Stockholm [19] Female patients and patients with previous P-Ca analysis were more likely to have a P-Ca analysis, which could be explained by women's greater risk of pHPT and recurrent check-ups of patients with chronic diseases.

In order to control for compositional confounding at the patient level we included an individual risk score for P-Ca-analysis. The inclusion of this variable did not explain the variation between physicians and between HCCs. Further, our empirical analysis found that the sex of the physician had no influence on P-Ca test ordering, in contrast to a study from Israel where female physicians ordered more test [20]. Older and more experienced physicians were less likely to order a P-Ca-test, which is in line with previous studies indicating that test ordering behaviour of GPs was influenced by years of experience [21]. P-Ca analyses done as part of group analyses used in surveillance of different chronic conditions may inflate the number of P-Ca analyses [22]. However, even though the number of group analyses was associated with higher frequency of P-Ca analysis, it could not explain the variation at the HCC level.

As explained in previous studies [23]; the measures of variation (e.g. median odds ratio) should be interpreted only for the specific time and place of the study, as there may be pattern of variance produced by different conditions. The associations, however, between characteristics of, on the one hand, the patients, physicians, and HCCs and on the other the frequency of P-Ca analysis, intend to provide information that can be generalised and applied to contexts beyond the one where the study was performed.

The risk for selection bias is low since this study is based on a large sample from a primary care area serving 97% of the population. Moreover, as this study is a retrospective database study, the ordering of analyses is not influenced by the study. A limitation of the study is that the frequency of ICD coded patient visits varies both between HCCs and according to diagnosis [9]. This might affect the risk score calculation.

Different views of the reason for screening could also affect the result. However national recommendations are well known in Swedish primary care [1,2] thus the risk for bias is minor. Due to regional variation in laboratory testing [8] the results from this study might not be applicable in all regions in Sweden.

In this study only the variables available in the SPCD database were included. In previous studies, other characteristics of the physician, such as attitude to risk taking and involvement in development of guidelines, explained parts of the higher level variance [8].

We found that there was variation between physicians and between HCC in ordering of P-Ca analysis, which is in line with previous studies [24]. However, in this study we also tried to quantify the contribution of each level by using the median odds ratio. Even though our multilevel approach identified factors, both at the physician and HCC level, which are important to consider for understanding the inclination to order a P-Ca test, none of the included variables could explain the variation at the higher level. The identification of yet unidentified factors that contribute to the variation is needed for monitoring of practice variation and quality assessment and for applying appropriate interventions to achieve optimal frequency of P-Ca analyses.

## Conclusions

National recommendations in Sweden have been issued to increase the frequency of P-Ca analyses to detect more patients with pHPT. There is a substantial variation in number of P-Ca analyses primarily between physicians but also between Health Care Centres. Female sex of the patient and increasing number of diagnoses is associated with higher propensity of P-Ca analysis. Physicians under education order most P-Ca analyses and locum least, but sex of the physician has no influence.

## Competing interests

The authors declare that they have no competing interests.

## Authors' contributions

SD conceived the study, drafted the manuscript, responded to the reviewer comments and critically revised the manuscript. PH conceived the study, participated on the design of the study, performed multilevel analyses and interpretation of data responded to the reviewer comments and critically revised the manuscript. HO participated on the design of the study, supported PH in the performance of the multilevel analyses and interpretation of data. RE conceived the study and critically revised the manuscript for important intellectual content. JM participated in the design of the study and interpretation of data and critically revised the manuscript for important intellectual content. KBB Conceived the study, drafted the manuscript, responded to the reviewer comments and critically revised the manuscript. All authors read and approved the final manuscript.

## Pre-publication history

The pre-publication history for this paper can be accessed here:

http://www.biomedcentral.com/1471-2296/11/43/prepub

## Supplementary Material

Additional file 1 **All the variables in the risk score equation**. We selected ICD-10 coded diagnoses and symptoms associated with pHPT. A risk score for a P-Ca analysis was created with stepwise logistic regression based on age, concomitant diagnosis and drug treatment. Total number of patients 154 629. Click here for file
