# Supplementary material for: Variation in plasma calcium analysis in primary care in Sweden - a multilevel analysis
Source: BMC Fam Pract. 2010 May 30;11:43. doi: 10.1186/1471-2296-11-43 (PMC2889884; doi:10.1186/1471-2296-11-43)
Supplement: Additional file 1 — All the variables in the risk score equation. We selected ICD-10 coded diagnoses and symptoms associated with pHPT. A risk score for a P-Ca analysis was created with stepwise logistic regression based on age, concomitant diagnosis and drug treatment. Total number of patients 154 629. [file 1471-2296-11-43-S1.DOC]

# Additional file

### Additional file to table 1 – All variables analysed for the risk score equation
